# Induction of Immunogenic Response in BALB/c Mice by Live and Killed Form of Recombinant *Lactococcus lactis* Displaying EG95 of *Echinococcus granulosus*

**DOI:** 10.52547/ibj.25.4.284

**Published:** 2021-06-30

**Authors:** Fatemeh Ebrahimzadeh, Hoda Shirdast, Amirhossein Taromchi, Yeganeh Talebkhan, Ali Haniloo, Abdolreza Esmaeilzadeh, Keivan Nedaei, Esmat Mirabzadeh

**Affiliations:** 1 Department of Medical Biotechnology, Faculty of Medicine, Zanjan University of Medical Sciences, Zanjan, Iran;; 2 Biotechnology Research Center, Pasteur Institute of Iran, Tehran, Iran;; 3 Department of Medical Parasitology and Mycology, Faculty of Medicine, Zanjan University of Medical Sciences, Zanjan, Iran;; 4 Department of Immunology, Faculty of Medicine, Zanjan University of Medical Sciences, Zanjan, Iran;; 5 Cancer Gene Therapy Research Center, Zanjan University of Medical Sciences, Zanjan, Iran;; 6 Department of Molecular Medicine, Biotechnology Research Center, Pasteur Institute of Iran, Tehran, Iran

**Keywords:** *Echinococcus granulosus*, * Lactococcus lactis*, *Immunization*, Vaccines

## Abstract

**Background:**

CE is a zoonotic parasitic infection caused by *Echinococcus granulosus* worldwide and is associated with economic losses among livestock animals*. *EG95 is an immunogenic antigen from the *E. granulosus.* *Lactococcus lactis *has been prested as a safe vehicle for antigen delivery. The goal of this study was to design a novel *L. lactis* strain displaying EG95 as a vaccine delivery system.

**Methods:**

The *eg*95 encoding gene fragment fused to the M6 anchoring protein was cloned into the pNZ7021 vector, and* L. lactis* NZ9000 displaying recombinant EG95 was constructed. The expression of an approximately 32-kDa EG95 protein was confirmed by Western blotting and immunofluorescence analysis. The immune responses were evaluated in BALB/c mice immunized orally and subcutaneously with the live and killed recombinant *L. lactis*, respectively.

**Results:**

Total IgG level in mice immunized with heat-killed recombinant *L. lactis *(pNZ7021-*eg*95) significantly increased compared to the control group. sIgA was significantly higher in mice received live recombinant *L. lactis *(pNZ7021-*eg*95) compared to the control mice. Splenic lymphocytes from immunized mice represented the high levels of IFN-γ and the low-levels of IL-4 and IL-10.

**Conclusion:**

Our results indicate that immunization with EG95-expressing *L. lactis *can induce both specific humoral and cellular immune responses in mice.

## INTRODUCTION

Cystic echinococcosis is a zoonotic infection caused by metacestode stage of the parasitic tapeworm, *Echinococcus granulosus*^[^^1^^]^. Two types of mammalian hosts are involved in the parasite’s life cycle. The definitive hosts are canids in which adult tapeworms can be developed in the small intestine, and the intermediate hosts are ungulates, including livestock (especially sheep), which are infected by the intake of the parasite eggs excreted in the feces of infected dogs and other canids. In addition, humans can be accidentally infected through the exposure to the parasite eggs. The ingested eggs hatch and release oncospheres that pass through the intestinal mucosa and frequently reach the liver and lungs via the portal and lymphatic vessels. Finally, they can develop as hydatid cysts in which many protoscoleces are produced asexually. When infected livestock offal is consumed by dogs or other canids, each protoscolex develops to an adult worm in the small intestine^[^^2^^]^. 

The high prevalence of the CE in many areas of the world influences public health and is considered as a main factor for remarkable economic losses among livestock animals, particularly in highly endemic regionss^[^^3^^]^. Currently, the control of CE mainly depends on the disposal of the infected offal, preventing dogs from feeding on infected livestock viscera and periodic treatment of dogs with praziquantel^[^^4^^]^. Moreover, vaccination of sheep has been recommended as a complementary and effective approach to control CE transmission by interrupting the tapeworm life cycle^[^^5^^-^^8^^]^

Several vaccine candidate antigens have been tested for the immunization against *E. granulosus*^[^^9^^-^^11^^]^. Among them, EG95 is considered as a protective antigen against CE in sheep. EG95 is developed as a recombinant vaccine and induces the high levels of protection (96-100%), when used as a vaccine in sheep as an intermediate host in New Zealand, Australia, and Argentina^[^^7^^,^^8^^]^. 

Probiotics are described as live bacteria having beneficial and positive influences on human beings and animal health. Accordingly, most of them are LAB, which are G-positive, non-colonizing, nonpathogenic, and food-grade microorganisms and mainly were exploited in the food industry for manufacturing fermented products due to a long history of their safe status^[^^12^^]^. LAB are normal microorganisms of the gastrointestinal tract, considered as "generally regarded as safe" with positive impact on the immune and digestion system^[^^12^^-^^14^^]^. Moreover, different LAB strains have been used as a safe expression system for the production and delivery of various proteins to treat diseases such as cervical cancer, diabetes, obesity, and inflammatory bowel diseases^[^^15^^-^^18^^]^. LAB can also be utilized as a live vector to deliver heterologous proteins to the mucosal surfaces, for vaccination and biomedical applications^[^^19^^-^^21^^]^.

Attractive and beneficial biological properties of LAB, including immunomodulatory and immuno-stimulation effects without inducing side effects, besides the adjuvanticity, ease of production, and suitable oral administration route, make them advantageous and effective candidates for vaccine development^[^^22^^,^^23^^]^. Therefore, several LAB such as *L. lactis* have been extensively exploited as a safe vehicle for the expression and delivery of foreign antigens, and their immunogenicity was evaluated for the development of vaccines against different infections^[^^24^^-^^26^^]^.

In most endemic developing countries, the production process and vaccination costs of sheep with the recombinant EG95 vaccine may prevent the vast utilization of the vaccine. Thus, in this study, we constructed a recombinant *L. lactis* NZ9000 to express surface displayed EG95 protein using the M6 cell wall anchor. The immune responses were evaluated by the immunization of BALB/c mice with live and heat-killed form of the recombinant *L. lactis* displaying EG95.

## MATERIALS AND METHODS

**Bacterial strains, media,** **and growth conditions **

*L. lactis *NZ9000 (MoBiTec, Germany) was cultivated at 30 ºC without shaking in M17 broth (Quelab, Canada) supplemented with 0.5% (w/v) glucose (GM17) and 10 µg/ml of chloramphenicol (Bio Basic, Canada). *Escherichia coli *TOP10 was cultured in Luria-Bertani medium (Sigma-Aldrich, USA) containing appropriate antibiotic (Bio Basic) on a shaker incubator at 37 ºC for 16 h.

**Gene synthesis**

A 972-bp gene fragment encoding the precursor protein SP_Usp45_-EG95-CWA_M6_, composed of the Usp45 signal peptide (the native lactococcal secretion signal sequence) fused to the *eg*95 coding sequence (GeneBank accession number X90928.1). Subsequently, the cell wall anchor fragment of the *Streptococcus pyogenes *M6 protein fused at its C-terminus was designed by ApE software and codon-optimized for protein expression in *L. lactis*. Moreover, *Sph*I and *Ban*II restriction sites were added to 5ꞌ and 3ꞌ ends of this construct. The synthesis of the gene sequence was performed in pGH cloning vector by the Generay Biotechnology Company (Shanghai, China). 

**Construction of the recombinant** ***L. lactis***

The bacterial strains, plasmids and primers utilized in the present study are listed in Tables 1 and 2. The *eg95* gene was PCR-amplified from the pGH-*eg*95 using M13 primers. The 1189-bp fragment was digested by *Ban*II and *Sph*I restriction enzymes (Thermo Fisher Scientific, USA), then was purified by CleanUp kit (GeneAll, Korea) and inserted into the *Ban*II/*Sph*I double-digested pNZ7021 (MoBiTec) by T4 DNA ligase (Thermo Fisher Scientific), resulting in pNZ7021-*eg*95. *L. lacis* strain NZ9000 was transformed by electroporation using a Gene Pulser (Eppendorf, Germany) in 0.1-cm electroporation cuvette (adjustment: 2 kV, 200 Ω, 25 μF). Following the electric pulse, the G-M17 broth was added to the mixture and incubated at 30 °C for 3 h. Finally, the NZ9000 transformants containing pNZ7021-*eg*95 were cultured on GM17 agar plates comprising 10 µg/ml of chloramphenicol and incubated at 30 °C for two days. Confirmation of the positive clones was performed by PCR utilizing PNZ primers. Extracted recombinant vectors were confirmed via restriction digestion and also sequencing.

**Table 1:** Bacterial strains and plasmids used in this study

**Strain or plasmid**	**Description**	**Source or references**
*E. coli *TOP10	Cloning host	Our lab
*E. coli *TOP10-pGH	*E. coli *Top10 containing pGH	This study
*L. lactis *NZ9000	MG1363 derivative, *pepN*::*nisRK*	^[^^27^^]^
*L. lactis *(pNZ7021)	*L. lactis *containing empty vector pNZ7021	This study
*L. lactis *(pNZ7021-* eg*95)	*L. lactis* containing pNZ7021-*eg*95	This study
pGH-*eg*95	pGH harboring *eg*95 gene	Generay Biotechnology
pNZ7021	*L. lactis* expression vector, Cm^R^, pNZ8148 derivative, pepN promoter, 3076bp	^[^^28^^]^
pNZ7021-*eg*95	pNZ7021 harboring *eg*95 gene	This study

**Polyclonal antibody** **production against the rEG95-GST protein**

Initially, 200 µg of rEG95-GST protein emulsified with Quil A adjuvant (gifted by Prof. Lightowlers, Australia) was administered intradermally to a female white rabbit. After two weeks, a booster immunization dose (200 µg) was given. Finally, two weeks later, the blood sample was obtained, and the serum sample was separated and stored at -80 ºC for further assays.

**Expression and characterization of the EG95 protein**

To express EG95 in *L. lactis*, the NZ9000 transformants harboring pNZ7021-*eg*95 were cultivated. The cells were centrifuged at 11,000 ×g for 10 min, disrupted by sonication and applied for Western blotting. The suspension was lysed in a sample buffer, and the bacterial protein supernatants were loaded on 12% SDS-PAGE. After transferring the proteins onto a nitrocellulose membrane, blocking the membrane was carried out with 5% skim milk in tris-*buffered* saline, 0.1% Tween 20 (TBST) at 4 °C overnight. Then the membrane was treated with rabbit polyclonal serum at 1:500 dilution for 2 h. The membrane was washed five times with TBST and incubated with 1:5000 HRP-conjugated goat anti-rabbit IgG secondary antibody (Sigma-Aldrich). Finally, color development was performed with DAB (Sigma-Aldrich).

**Immunofluorescence analysis**

*L. lactis *NZ9000 cells were cultivated, centrifuged at 2800 ×g for 10 min and resuspended in PBS. The bacterial pellet was placed on Poly-L-lysine-coated glass slides and incubated at RT for 20 min. The fixation of the slides was performed with 4% paraformaldehyde. Next, the cells were blocked with 4% BSA (Sigma-Aldrich) in PBS for 30 min. The fixed cells were then incubated with 1:250 diluted rabbit anti-EG95 polyclonal serum at RT for 90 min and then washed three times with PBS. The slides were incubated with a 1:1000 FITC-conjugated goat anti-rabbit antibody (Sigma-Aldrich) for 90 min and washed three times with PBS at RT. Finally, the labeled slides were mounted with glycerol in PBS (pH 8.5-9.0) and then analyzed by immunofluorescence microscopy (Olympus, Japan).

**Mice immunization**

**Fig. 1:**
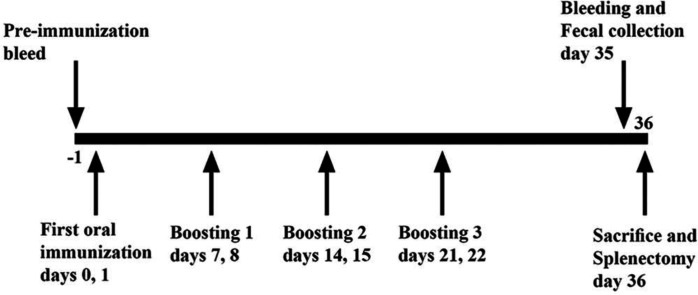
Schematic representation of the oral immunization, serum, and fecal sampling schedule. Three groups of BALB/c mice were orally immunized on days 0 and 1 and received boosters on days 7 and 8, 14 and 15, and 21 and 22. Blood and fecal samples were collected on day 35. The mice were sacrificed on day 36

**Fig. 2.**
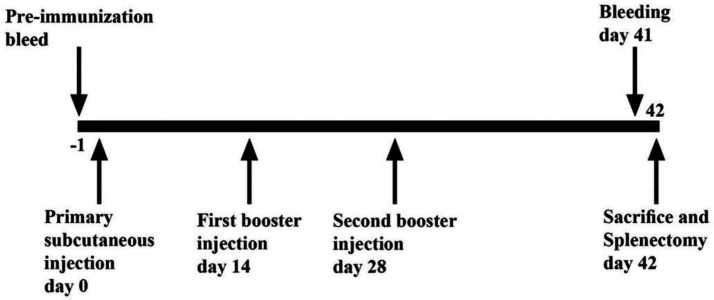
Schematic representation of subcutaneous immunization and serum collection schedule. Four groups of BALB/c mice subcutaneously immunized on days 0, 14, and 28. Blood samples were collected on day 41. On day 42, all the mice were sacrificed

Pathogen-free female BALB/c mice (6-8 weeks old) were purchased from Razi Vaccine and Serum Research Institute (Iran), housed and acclimatized for one week in the Animal Care Facility Unit of Medicine in Zanjan University of Medical Sciences under a 12-h light/12-h dark schedule with free access to food and water. Mice were divided into seven groups (n = seven/group). Two groups of mice were orally immunized with 10^10^ CFU^[^^29^^,^^30^^]^ of live *L. lactis* NZ9000 strains containing pNZ7021-*eg*95 vector, as the test group and *L. lactis* NZ9000 bacterium harboring pNZ7021 empty vector as a control group in 200 µl of sterile PBS after fasting conditions for 8-10 h. As a negative control group, PBS was administered orally. For the oral administration, mice received eight treatments in four weeks. Two other groups were immunized subcutaneously with 10^10^ CFU of heat-killed *L. lactis* NZ9000 strains on days 0, 14, and 28. The test and control groups were administered with heat-killed recombinant *L. lactis *containing pNZ7021-*eg*95 vector and *L. lactis* with empty pNZ7021 vector, respectively. Killed bacteria were prepared by heating at 60 °C for 20 min. As the positive and negative control groups, purified rEG95-GST protein (20 µg) emulsified with Quil A adjuvant and PBS were administered subcutaneously to mice on days 0, 14, and 28. The mice were bled on day 0 (before immunization) for obtaining pre-immunized sera and two weeks after the last immunization. Blood samples were collected, and serum samples were separated and kept at -80 ºC for ELISA test. The animal experiment procedures are demonstrated in Figures 1 and 2.

**Table 2:** Primers used in this study

**Primers**	**Forward 5´ to 3´**	**Reverse 5´ to 3´**	**Description**
M13	GTTTTCCCAGTCACGAC	GCGGATAACAATTTCACACAGG	pGH gene construct
PNZ	TGGGAATCATCACGTTCAGGT	GGCTATCAATCAAAGCAACACG	PNZ7021 ligation test

**Cytokine measurement and** ***in vitro*** **splenocyte proliferation assay**

Two weeks after the last immunization, five mice in each group were killed, and their spleens were aseptically removed. The splenocytes were isolated by single-cell suspension preparation via mechanical dissociation and homogenization. After red blood cells lysis by ACK solution (150 mM NH4Cl, 0.1 mM Na2EDTA, 10 mM KHCO3) and three washes with PBS, the splenocytes were resuspended in RPMI 1640 medium (Inoclon, Iran) containing 10% heat-inactivated fetal bovine serum (*Gibco,* *UK*), 2 mM of L-glutamine, 0.05 M of 2-mercaptoethanol, and 1% penicillin-streptomycin (Sigma-Aldrich) and counted. Afterwards, 3 × 10^6^ cells/ml were plated with purified rEG95 (10 µg/ml), ConA (5 mg/ml; Sigma-Aldrich), as the positive control and without any additive as a negative control and incubated with 5% CO_2_ at 37 °C for 72 h. The supernatants were then collected for the measurement of IFN-γ, IL-10, and IL-4 levels using available ELISA kits (R&D Systems, USA) according to the manufacturer's protocols. The proliferative response of splenocytes was determined by MTT assay. After removing the supernatants, 20 µl of MTT (5 mg/ml; Sigma-Aldrich) was added to each well and incubated at 37 °C for 4 h. The plate was centrifuged at 1,000 ×g 22 °C for 5 min and then was dissolved in 150 µl of DMSO (Merck, Germany) at 37 ºC for 20 min. The optical absorbance was measured at 570 nm with a reference wavelength of 630 nm by an ELISA microplate reader (BioTek, USA). Calculation of proliferative index as the definition of the proliferative responses was performed as follows: the average absorbance of the stimulated culture/the average absorbance of the non-stimulated culture.

**Determination of humoral immune responses by ELISA**

Mouse EG95-specific antibodies were determined using purified rEG95 as an antigen by ELISA. The 96- well plates (Greiner Bio-One) were coated with 0.05 ng/ml of purified rEG95 protein at 4 ºC overnight. The plates were then washed three times with PBS containing 0.05% Tween 20 (PBST) and blocked with PBS containing 1% BSA at RT for 1 h. After three washes with PBST, 1:200 dilution of serum samples (100 µl/well) was added in duplicate as primary antibodies and incubated at RT 2 h. The plates were washed three times with PBST. For total IgG assay, 1:1000 dilution of an HRP-conjugated polyclonal goat anti-mouse IgG (Bio-Rad) was added at RT for 1 h. 

Measurement of IgG1, IgG2, IgG3, IgM, and IgA antibodies was performed by adding 100 µl/well of an anti-isotype monoclonal antibody (Sigma-Aldrich) specific for each antibody class for one hour. After washing the plates, 1:15000 HRP-conjugated rabbit anti-goat IgG (Sigma-Aldrich) was added to each well at RT for 1 h. In each assay, after three times washing with PBST, 100 µl of ABTS peroxidase substrate (KPL, SeraCare Life Sciences, USA) was added to each well, and the enzymatic reaction was stopped by the addition of 1% SDS. The optical absorbance was measured at 405 nm using an ELISA reader (BioTek). Fresh fecal pellets were collected two weeks after the last immunization from mice orally immunized with live *L. lactis*, resuspended in PBS containing 1% BSA, and incubated at 4 °C overnight. The suspensions were vortexed, centrifuged at 18,000 ×g at 4 ºC for 10 min, and the supernatants were used to measure sIgA levels using ELISA. Briefly, 96-well plates were coated with 0.1 ng/ml of purified rEG95 protein at 4 ºC overnight. The plates were then washed three times with PBST, and the wells were blocked with BSA and incubated at RT for 1 hour. After washing three times with PBST, 100 µl of fecal suspension was added to each well and incubated at RT for 2 h. sIgA was detected using a goat monoclonal anti-mouse IgA and an HRP-conjugated rabbit anti-goat IgG (both from Sigma-Aldrich).

**Statistical analysis**

Statistical analysis of the data was accomplished using GraphPad Prism 8 (GraphPad, CA). The results were compared using one-way analysis of variance (ANOVA) and were represented as the mean ± SD. The *p *values <0.05 were considered as statistically significant levels.

**Ethical **statement

All animal handlings followed the ethical standards of the Animal Research Ethics Committee, Zanjan University of Medical Sciences, Zanjan, Iran (ethical code: ZUMS.REC.1396.64).

## RESULTS

**Plasmid construction and expression of EG95 protein in** ***L. lactis***

**Fig. 3:**
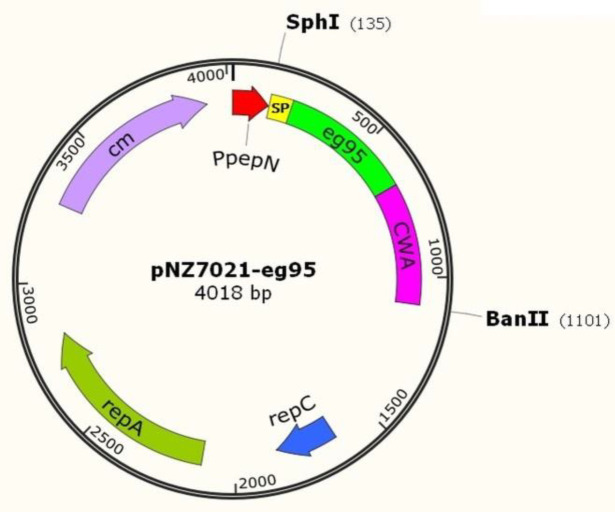
The schematic view of the recombinant expression vector pNZ7021-*eg*95. PpepN, constitutive promoter; SP, Usp45 signal peptide sequence; eg95, *eg*95 gene coding sequence; CWA, M6 cell-wall anchor of S*treptococcus pyogenes*; rep C and rep A, replication gene C and A, respectively; Chol, chloramphenicol resistance gene

**Supplementary Fig. S1:**
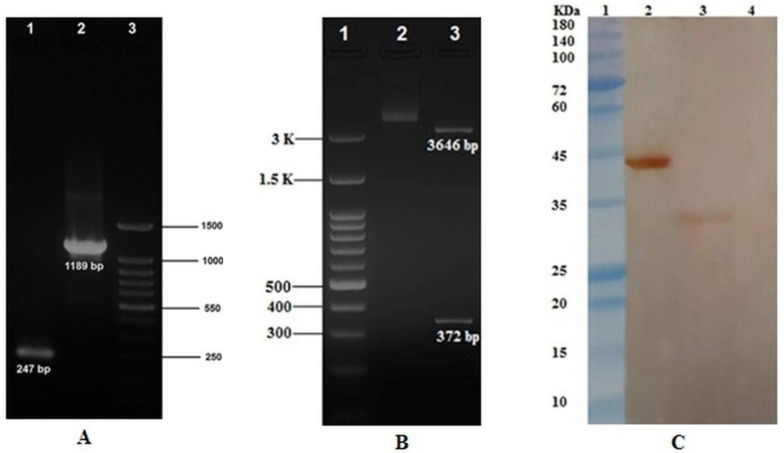
Identification of recombinant plasmid pNZ7021-*eg*95 and detection of the EG95 protein expression. (A) PCR amplification of *eg*95 fragment. Lane 1, PCR product from empty pNZ7021 vector (247 bp); lane 2, PCR product from recombinant pNZ7021-*eg*95 vector (1189 bp); lane 3, DNA ladder (100 bp). (B) Restriction digestion analysis of the pNZ7021-*eg*95 expression vector. Lane 1, DNA ladder (100 bp); lane 2, undigested pNZ7021-*eg*95 vector; lane 3, *Bgl*II digested pNZ7021-*eg*95 expression vector. (C) EG95 identity confirmation through Western blotting. Lane 1, protein MW marker; lane 2, purified rEG95-GST protein (43.5 kDa; positive control); lane3, recombinant *L. lactis* NZ9000 strain containing pNZ7021-*eg*95 vector (~33 kDa); lane 4, *L. lactis* NZ9000 strain containing empty pNZ7021 plasmid (negative control).

The graphic view of the pNZ7021-*eg*95 plasmid designed by SnapGene version 1.1.3 software (GSL Biotech LLC, CA, USA) is shown in Figure 3. The recombinant expression vector was verified by PCR amplification using PNZ primers, specifically designed for the flanking regions of the multiple cloning sites of plasmid backbone. The expected amplicon size was 1189 bp (Supplementary Fig. S1A). Restriction digestion also confirmed the cloning procedure (Supplementary Fig. S1B). Immunoblotting with polyclonal antiserum against purified rEG95-GST confirmed an approximately 33 kDa protein consistent with the size of precursor SP_Usp45_- EG95-CWA_M6_, in the total protein extract of the recombinant *L. lactis* (Supplementary Fig. S1C).

**Surface expression of the EG95**

**Fig. 4:**
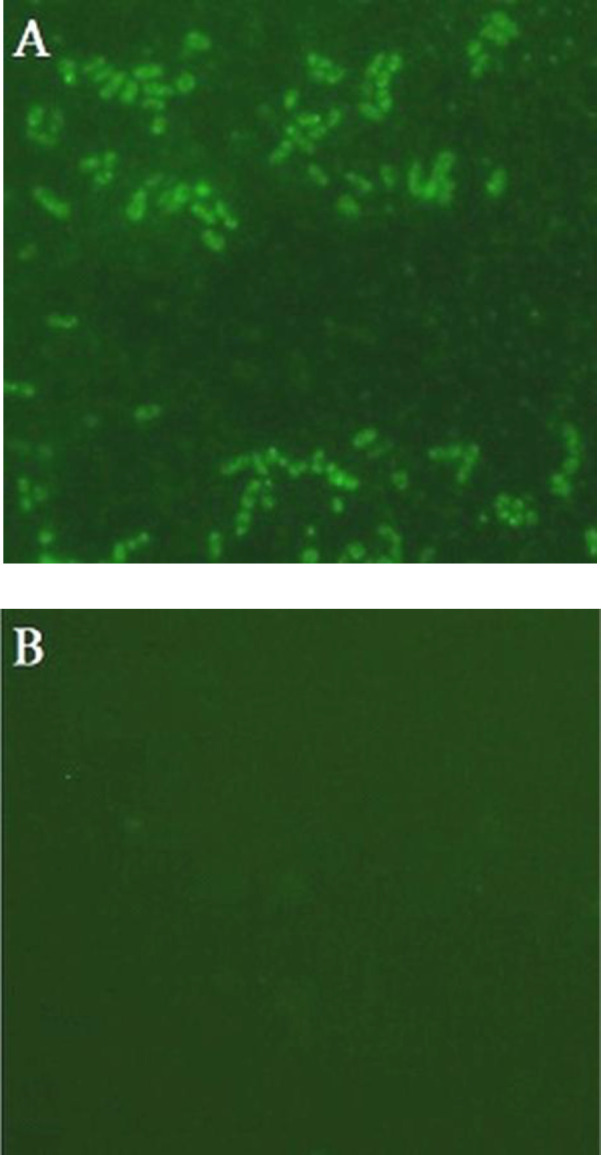
Detection of recombinant EG95 protein on the surface of *L. lactis* by immunofluorescence analysis. (A) Recombinant *L. lactis* NZ9000 cells expressing EG95-CWA_M6; _(B) *L. lactis* NZ9000 cells containing empty pNZ7021 vector (negative control).

The immunofluorescence assay performed to confirm that recombinant *L. lactis *strain NZ9000 displayed EG95 protein on the cell surface. Recombinant *L. lactis* NZ9000 (pNZ7021-*eg*95) cells revealed fluorescence (Fig. 4A), which was significantly different from the control strain NZ9000 (pNZ7021; Fig. 4B). 

**Assessment of antibody responses**

**Fig. 5.**
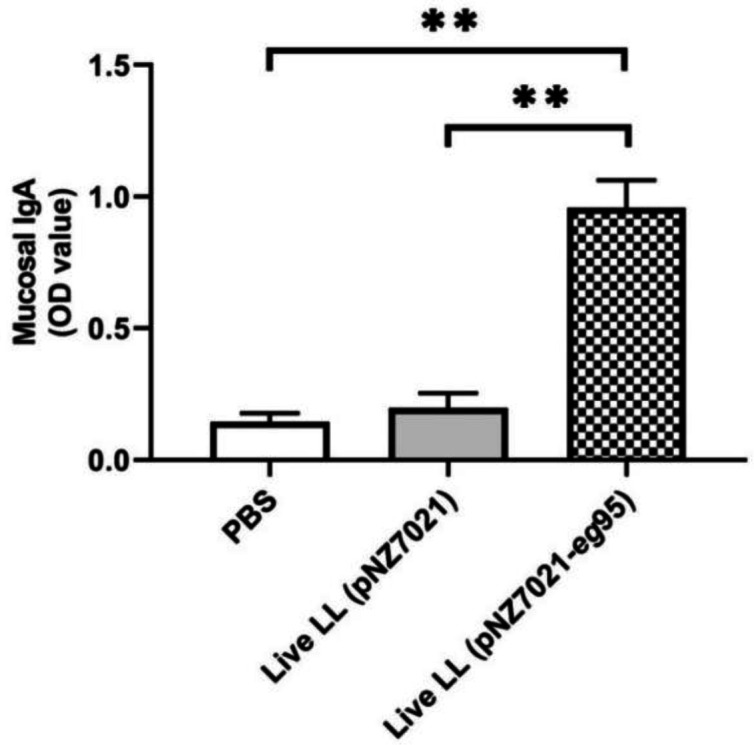
Anti-EG95 sIgA antibody responses in mice orally immunized with PBS, *L. lactis *(pNZ7021), and live recombinant *L. lactis *(pNZ7021-* eg*95). Fecal samples were assessed for EG95-specific IgA by ELISA. Data are shown as mean ± SD of duplicate experiments

**Fig. 6:**
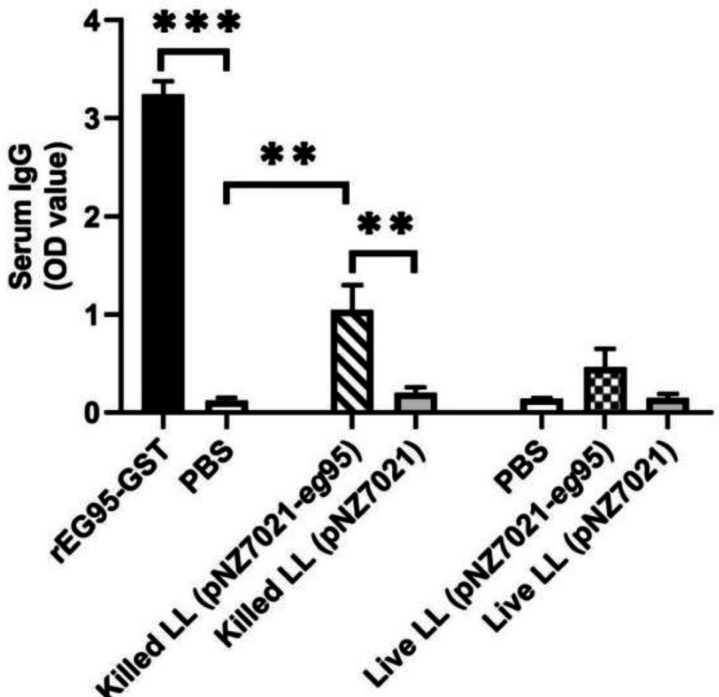
Detection of EG95-specific total IgG in serum samples of all experimental BALB/c groups. A total of five mice from each group were tested in the ELISA test. Data are shown as mean ± SD of duplicate experiments

**Supplementary Fig. S2:**
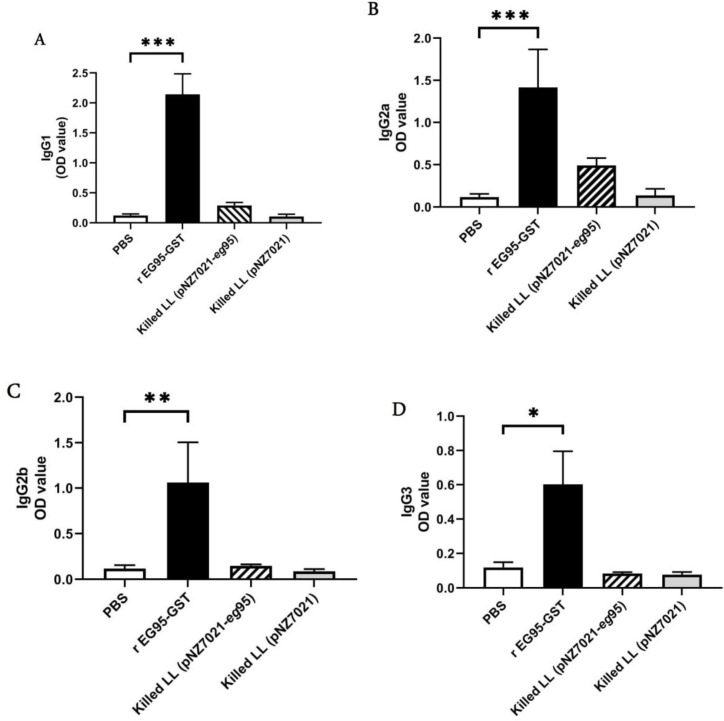
Assessment of EG95-specific IgG subclasses in mice subcutaneously immunized with PBS and killed recombinants *L. lactis *(pNZ7021-*eg*95) and *L. lactis *(pNZ7021). IgG1 (A), IgG2a (B), IgG2b (C), and IgG3 (D) levels were measured by ELISA. Data are shown as mean ± SD of duplicate experiments

sIgA levels in mice orally received live *L. lactis *(pNZ7021-*eg*95) was significantly higher than the mice immunized with live *L. lactis *(pNZ7021) and PBS control group (*p* < 0.05, Fig. 5). Assessment of total serum IgG levels in all tested groups is represented in Figure 6. The total IgG level in mice immunized with heat-killed recombinant *L. lactis* (pNZ7021-*eg*95) was significantly increased compared to those immunized with heat-killed *L. lactis *(pNZ7021) and PBS control group (*p* < 0.01). There were no significant differences of the live *L. lactis *(pNZ7021-*eg*95) and live *L. lactis *(pNZ7021) with the PBS control group for IgG production (*p *> 0.05). The mice immunized with the purified rEG95 showed a significant IgG response compared to the PBS control group (*p *< 0.001). The assessment results of IgG subclasses (IgG1, IgG2a, IgG2a, and IgG3) demonstrated that the levels of IgG1 and IgG2a antibodies increased, but no significant differences were observed for IgG subclasses between test and control groups (*p *> 0.05, Supplementary Fig. S2). Moreover, there were no significant differences between test and control groups for serum IgA and IgM isotypes (*p *> 0.05, data not shown).

**Proliferative responses of splenocytes**

**Fig. 7:**
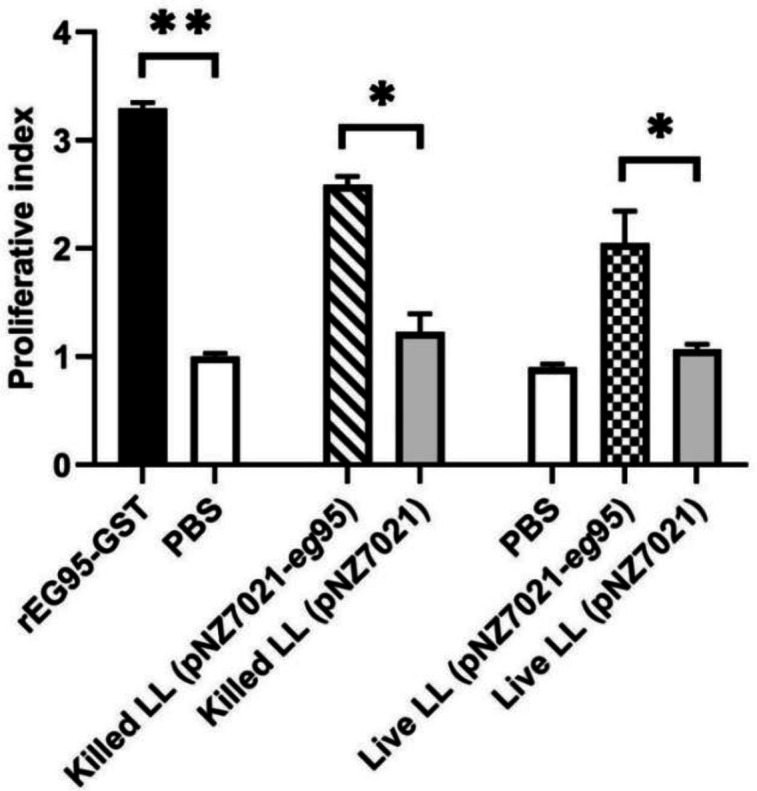
Proliferative responses of murine splenocytes stimulated with EG95 antigen *in vitro*. The MTT assay was used to analyze splenocyte proliferation response towards EG95 antigen following 72-h stimulation with 10 µg/ml of purified rEG95 or 5 mg/ml of ConA as T cell mitogenic agent. The bars represent the mean OD values of splenocytes proliferation of immunized mice in each group

As demonstrated in Figure 7, the mice immunized with killed and live recombinant* L. lactis* (pNZ7021-*eg*95) demonstrated a significant T-cell proliferative response compared to the mice immunized with killed and live* L. lactis *(pNZ7021) and PBS control group (*p *< 0.05). The mice immunized with purified rEG95 showed a significant T-cell proliferative response compared to the PBS control group (*p *< 0.01).

**Cytokine assay**

**Fig. 8:**
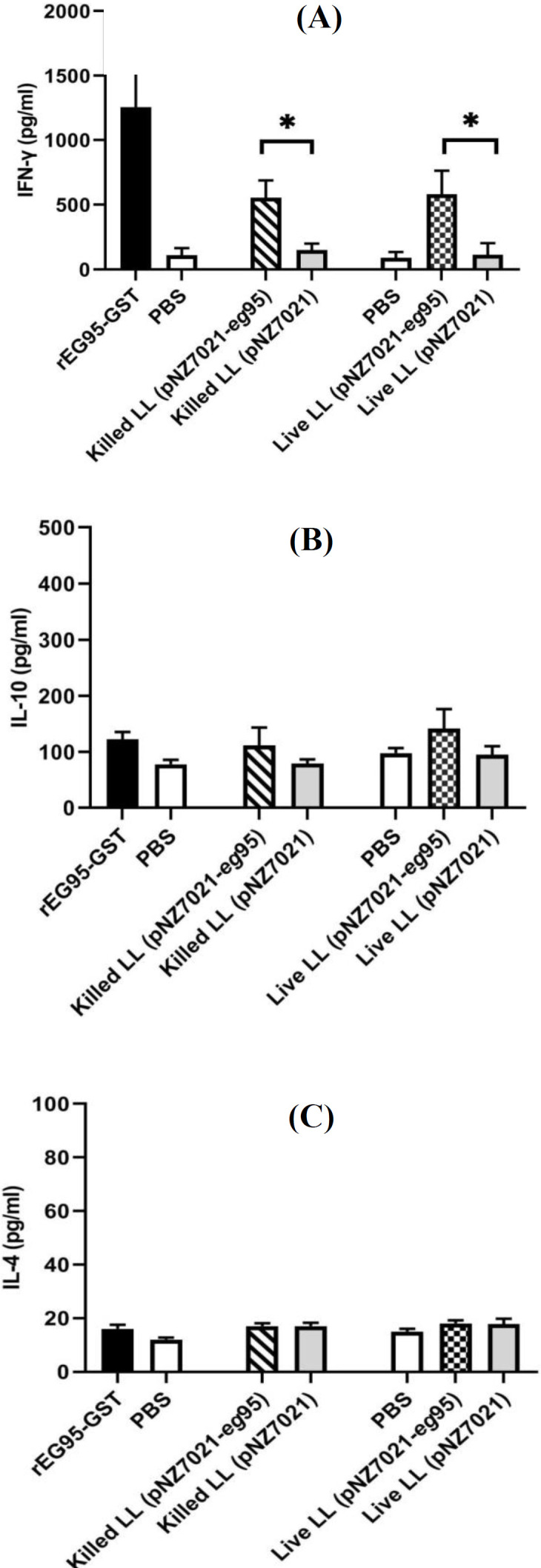
Assessment of cytokine production in murine splenocytes stimulated with EG95 antigen *in vitro*. The splenocytes isolated from mice immunized with *L.* *lactis* strains or purified rEG95 protein were stimulated with 10 µg/ml of purified rEG95 or 5 mg/ml of ConA. Supernatants were collected after 72 h of stimulation. IFN-γ (A), IL-10 (B), and IL-4 (C) levels were measured by ELISA. Data are shown as mean ± SD of duplicate experiments (n = 5).

The mice immunized with live and killed recombinant* L. lactis* (pNZ7021-*eg*95) showed the higher levels of IFN-γ compared to the mice immunized with the live and killed* L. lactis *(pNZ7021) and PBS control group (*p *< 0.05, Fig. 8A). IFN-γ levels in the mice immunized with purified rEG95 were significantly higher than those immunized with the PBS control group (*p *< 0.01, Fig. 8A). However, there were no significant differences between all test and control groups for the production of IL-10 and IL-4 (*p *> 0.05, Fig. 8B and 8C). 

## DISCUSSION

LAB expressing heterologous antigens is an attractive platform for vaccine delivery. Recombinant LAB was represented to stimulate systemic and mucosal immune responses^[^^23^^]^. Studies have shown that heat-killed probiotics, similar to live microorganisms, have beneficial effects on the host^[^^31^^-^^33^^]^. There are some reports on the successful trials of the surface antigen displaying *L. lactis* as vaccine carriers^[^^25^^,^^26^^]^. This form of expression has been considered advantageous for having better recognition by the immune system and probably evokes more robust immune responses. Therefore, the surface display in the LAB can be ideal and beneficial for vaccination compared to other forms^[^^34^^-^^36^^]^. For anchoring EG95 protein to the cell surface, we utilized Usp45 signal peptide at the N-terminal and the M6 cell-wall anchor at the C-terminal of the protein, and our results showed that EG95 could effectively be exhibited at the cell wall of *L. lactis *(Fig. 4).

The oral route of immunization can induce the mucosal immune responses; however, it is not necessarily effective as the subcutaneous route in the induction of systemic immunity. For determining the effective type of immunity, administration of live and heat-killed *L. lactis* bacteria to mice groups was performed as an immunization schedule.

Different antibody responses, including specific IgG subclasses, have been shown in parasitic infections^[^^37^^,^^38^^]^. Immunization of mice and sheep with oncosphere antigens, hydatid cyst fluid, and protoscoleces of *E. granulosus* could induce IgG, IgG1, and IgG2a responses^[^^39^^-^^41^^]^. Sheep vaccination using the EG95 vaccine in experimental trials produced the high levels of total IgG, IgG1 and IgG2 antibodies^[^^42^^]^. The specific anti-EG95 IgG and IgG2a antibodies increased by the EG95 DNA vaccination of BALB/c mice^[^^43^^]^. In our study, although the total IgG level in mice immunized with heat-killed *L. lactis* significantly increased compared to the control group, no significant differences were observed for IgG subclasses between the test and control groups. Moreover, serum IgG titers differed significantly between the mice immunized with the purified rEG95 and the mice vaccinated with killed and live recombinant *L. lactis* (pNZ7021-*eg*95).

We observed that the oral immunization of mice with live r*L.lactis*/pNZ7021-*eg*95 could lead to EG95-specific sIgA production; however, it could not induce IgG antibody. On the other hand, subcutaneous immunization with heat-killed *L. lactis *expressing EG95 induced higher levels of IgG. Heat-killed r*L.lactis*/pNZ7021-*eg*95 also showed another potential of this platform in vaccine designing without next downstream expensive preparation such as purification of protein. We examined this alternative form besides the oral live form, and the results showed that this model's systemic immune response was significant. Earlier studies have reported that the induction of IgG antibody against EG95 antigen correlates with protection against challenge infection with *E. granulosus* eggs in sheep that had been vaccinated with the EG95 vaccine^[^^44^^]^. On the other hand, oral immunization with the recombinant *L. lactis *expressing EG95 antigen can elevate mucosal immune responses (sIgA) and may confer intestinal protection against oncospheres in intermediate hosts infected by ingestion of parasite eggs. In general, regarding the infections that occur via the mucosal sites, an effective vaccine should be able to stimulate both mucosal and systemic immune responses^[^^45^^]^. Accordingly, the induction of sIgA and serum IgG implies that the co-administration of two forms of the vaccine may be more effective against CE in intermediate hosts.

Following immunization with *L. lactis*-expressing EG95, the splenocytes were stimulated with rEG95 protein, and then splenocyte proliferation and cytokine production were evaluated. The results of our study indicated that the proliferative response in the mice group immunized with live and heat-killed r*L.lactis*/pNZ7021-*eg*95 remarkably increased compared to the control group. This result confirms the specific induction of cellular proliferation by the EG95 antigen. Also, we showed that the production of IFN-γ was notably increased in both mice groups immunized with live and heat-killed r*L.lactis*/pNZ7021-*eg*95 compared to the control groups. Moreover, IFN-γ levels differed significantly between the mice immunized with the purified rEG95 and those vaccinated with killed and live recombinant *L. lactis* (pNZ7021-*eg*95). However, IL-10 and IL4 levels were not statistically different between the immunized and control groups. An increase in the IFN-γ/IL-10 ratio in the immunized mice may be a sign of protective immune responses. IFN-γ demonstrates immuno-modulatory effects by increasing antigen processing and presentation. An essential role of IFN-γ is to identify and eliminate pathogens. Production of IFN-γ also involved in Th1 differentiation^[^^46^^]^. Previous study have revealed both Th1 and Th2 immune responses in the human infected with hydatid cyst^[^^47^^]^. A Th1 response relates to protective immunity, whereas a Th2 response results in CE susceptibility^[^^48^^,^^49^^]^. Th1 and Th2 immune responses have been demonstrated in the mice immunized with the EG95 genetic vaccine and then infected with *E. granolosus *protoscoleces. The level of Th1 cytokines significantly increased, while that of Th2 cytokines decreased after vaccination with the EG95 vaccine. A predominant Th1 response was associated with protection against secondary hydatidosis^[^^50^^]^.

It should be noted that due to the effective impact of the EG95 antigen on the oral route, its permanent use in livestock feeds and even feeding dogs with other protoscoleces-specific antigens by *L. lactis* delivery system, as a simple strategy for the creation of immunity in livestock and dogs, seems promising but more studies are needed to be conducted. It is also necessary to evaluate the immunogenicity and immunoprotection of recombinant *L. lactis*-expressing EG95 among intermediate hosts such as sheep by their challenging with *E. granulosus* eggs per oral or more. In this work, we performed a novel presentation of *E. granulosus* EG95 at the surface of *L. lactis* as an antigen carrier vehicle. Our results support that *L. lactis* expressing cell-surfaced EG95 antigen can be utilized as a new vaccine candidate against cystic hydatid disease.
